# The haemagglutinin–neuraminidase protein of velogenic Newcastle disease virus enhances viral infection through NF-κB-mediated programmed cell death

**DOI:** 10.1186/s13567-024-01312-y

**Published:** 2024-05-07

**Authors:** Xiaolong Lu, Tiansong Zhan, Qiwen Zhou, Wenhao Yang, Kaituo Liu, Yu Chen, Ruyi Gao, Jiao Hu, Min Gu, Shunlin Hu, Xin-an Jiao, Xiaoquan Wang, Xiufan Liu, Xiaowen Liu

**Affiliations:** 1 Animal Infectious Disease Laboratory, College of Veterinary Medicine, Yangzhou University, Yangzhou, 225000 China; 2 Jiangsu Coinnovation Center for Prevention and Control of Important Animal Infectious Diseases and Zoonosis, Yangzhou University, Yangzhou, 225000 China; 3 Jiangsu Key Laboratory of Zoonosis, Yangzhou University, Yangzhou, 225000 China

**Keywords:** Newcastle disease virus, haemagglutinin–neuraminidase, viral infection, programmed cell death, NF-κB

## Abstract

The haemagglutinin–neuraminidase (HN) protein, a vital membrane glycoprotein, plays a pivotal role in the pathogenesis of Newcastle disease virus (NDV). Previously, we demonstrated that a mutation in the HN protein is essential for the enhanced virulence of JS/7/05/Ch, a velogenic variant NDV strain originating from the mesogenic vaccine strain Mukteswar. Here, we explored the effects of the HN protein during viral infection in vitro using three viruses: JS/7/05/Ch, Mukteswar, and an HN-replacement chimeric NDV, JS/MukHN. Through microscopic observation, CCK-8, and LDH release assays, we demonstrated that compared with Mukteswar and JS/MukHN, JS/7/05/Ch intensified the cellular damage and mortality attributed to the mutant HN protein. Furthermore, JS/7/05/Ch induced greater levels of apoptosis, as evidenced by the activation of caspase-3/8/9. Moreover, JS/7/05/Ch promoted autophagy, leading to increased autophagosome formation and autophagic flux. Subsequent pharmacological experiments revealed that inhibition of apoptosis and autophagy significantly impacted virus replication and cell viability in the JS/7/05/Ch-infected group, whereas less significant effects were observed in the other two infected groups. Notably, the mutant HN protein enhanced JS/7/05/Ch-induced apoptosis and autophagy by suppressing NF-κB activation, while it mitigated the effects of NF-κB on NDV infection. Overall, our study offers novel insights into the mechanisms underlying the increased virulence of NDV and serves as a reference for the development of vaccines.

**Supplementary Information:**

The online version contains supplementary material available at 10.1186/s13567-024-01312-y.

## Introduction

Newcastle disease (ND), caused by the Newcastle disease virus (NDV), results in significant damage and substantial economic losses within the global poultry industry [1]. According to the most recent virus taxonomy, NDV is classified within the Orthoavulavirus genus of the Paramyxoviridae family [2]. Generally, NDV can be categorized into multiple pathotypes, including lentogenic (low virulence), mesogenic (moderate virulence), and velogenic (high virulence) [3]. The haemagglutinin–neuraminidase (HN) protein is located on the envelope of viral particles, mediating the notable biological activity of NDV [4, 5]. This membrane protein can exert a significant regulatory influence on the pathogenicity and infection of NDV [6]. Notably, both amino acid (aa) variation and complete HN replacement can impact virus replication and pathogenicity [7, 8].

Programmed cell death is a deliberate and organized form of cell death aimed at maintaining homeostatic stability in response to external or internal signals [9]. As a crucial component of programmed cell death, autophagy is essential for preserving cellular homeostasis by enabling the breakdown of impaired organelles, surplus proteins, and invading microbes [10]. Moreover, apoptosis is an important type of programmed cell death marked by caspase hydrolysis and activation, as well as signal transmission. It is delineated by two interrelated signalling cascades (the extrinsic and intrinsic pathways), which operate independently. Several caspases, such as caspase-3/8/9, can be stimulated to trigger and regulate apoptosis [11]. NDV has been widely confirmed to affect both autophagy and apoptosis. With respect to autophagy, the combined action of NDV HN and F synergistically triggered autophagic flux by activating the AMPK–mTORC1–ULK1 pathway in vitro [12]. The induction of autophagy by NDV P and NP can be controlled through the PERK/ATF6 signalling pathway. Knocking down the P and NP genes can effectively suppress NDV replication in vitro [13]. The NDV HN protein can induce apoptosis in concert with TNF-α by activating both the intrinsic and extrinsic pathways in vitro [14]. Notably, a heightened apoptotic rate is correlated with the severity of the disease induced by NDV [15].

NF-κB is a transcription factor that governs various cellular processes, including cell apoptosis and autophagy. Generally, NF-κB exerts antiapoptotic effects, whereas it serves as a double-edged sword for autophagy, with different effects depending on the extracellular environment [16–18]. The activation of NF-κB can be regulated by IκB-α, which forms a complex with NF-κB in the absence of NF-κB activation. However, upon external stimulation of host cells, IκB-α may undergo phosphorylation followed by ubiquitination-mediated degradation. The nuclear localization sequences of NF-κB are exposed following the dissociation of IκB-α, which enables the transport of the complex into the cell nucleus where NF-κB regulates transcription [19].

Previous studies have shown an evolutionary transition of the mesogenic NDV strain Mukteswar into the velogenic NDV strain JS/7/05/Ch. A mutation in the HN protein is a crucial factor in the enhanced virulence and pathogenicity of JS/7/05/Ch [20]. However, the impact of the mutant HN protein on in vitro infection by NDV remains unclear. In this study, we employed three model viruses to examine the in vitro function of the mutant HN protein. As our study demonstrated, the velogenic variant NDV strain JS/7/05/Ch exhibited increased cellular damage and mortality attributed to the mutant HN protein. Further investigation revealed the crucial role of the mutant HN protein in triggering apoptosis and autophagy through activation of NF-κB. Moreover, the mutant HN protein attenuated the reciprocal effects of NDV infection on NF-κB activity. This study is the first to clarify the involvement of the NDV HN protein in programmed cell death via the NF-κB pathway. These findings provide essential insights into the exact mechanism underlying the diverse pathogenicity of genotype III NDVs.

## Materials and methods

### Viruses, cells, and homology modelling

**Figure 1:**
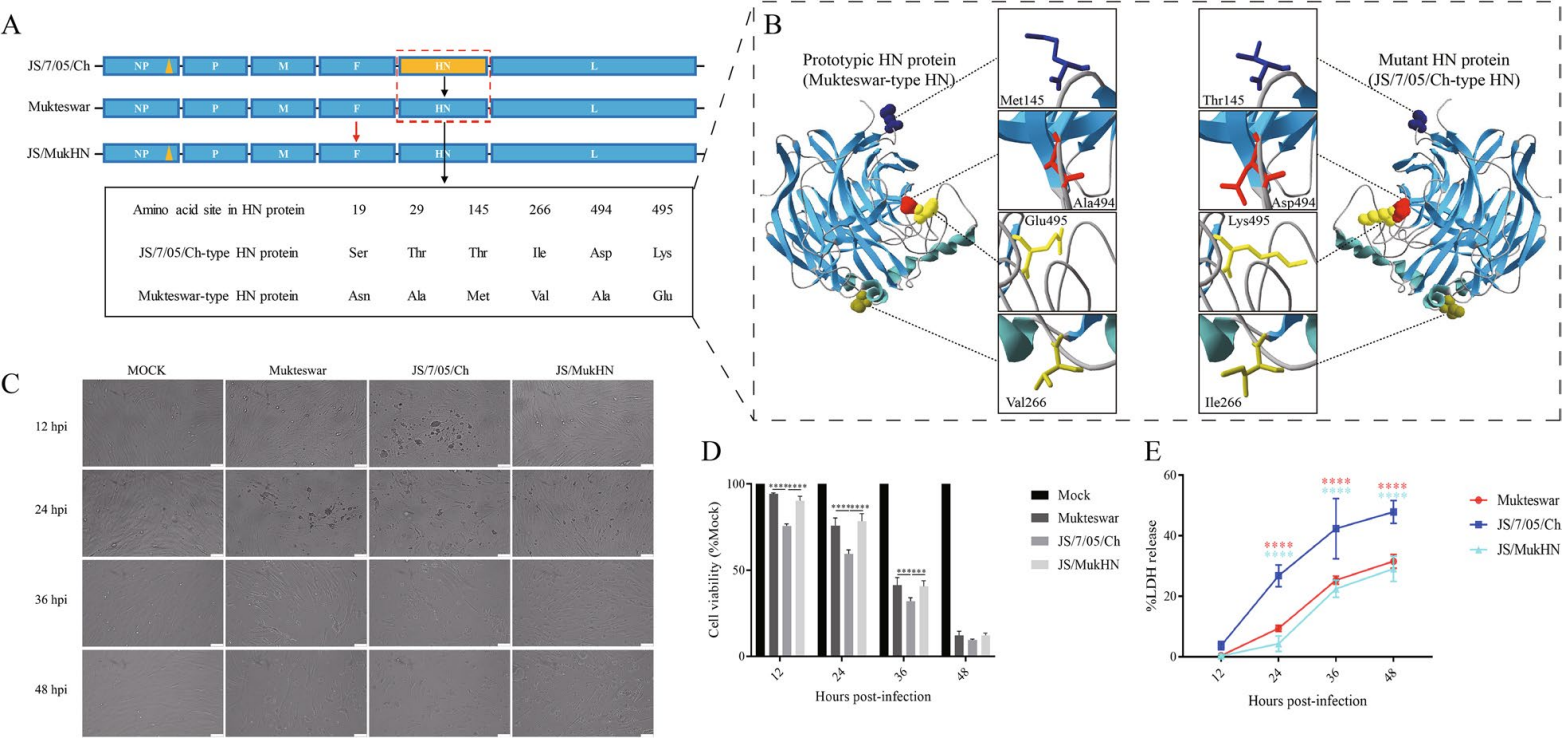
**Schematic representation of the NDVs utilized in this study, along with the cytopathic damage induced by these NDVs.** **A** A schematic representation illustrating the process of constructing the NDVs. The JS/MukHN strain is derived from JS/7/05/Ch and incorporates the Mukteswar-type HN gene through an NDV reverse genetics system. The HN proteins of the two parental viruses exhibited variations at six distinct aa sites: Asn 19 Ser, Ala 29 Thr, Met 145 Thr, Val 266 Ile, Ala 494 Asp, and Glu 495 Lys. Additionally, there is an aa mutation (Pro 438 Ser) in the NP protein in one of the parental viruses. The aa mutation in the NP protein of JS/7/05/Ch is indicated by an orange triangle, while its mutant HN protein is highlighted in an orange box. **B** The automated mode of SWISS-MODEL and PyMol 2.4.0 software were utilized to generate homology models of the HN proteins, employing a matching PDB file (ID: 1e8v.1. **A**) as previously described [20]. The model NDV strains express diverse HN proteins, and four differential aa sites (Met145Thr, Val266Ile, Ala494Asp, and Glu495Lys) between the prototypic and mutant HN proteins lead to modifications in the three-dimensional protein structure. The spatial locations of the four mutations are depicted in the zoom-in picture, and the distinct aa sites are emphasized using various colours. **C** The morphology of the infected CEFs was visualized under a microscope (100×). Scale bar: 50 μm. **D** The viability of infected CEF cells was determined with a CCK-8 detection kit. (E) LDH release levels were quantified in the cell culture supernatants of the NDV-infected cells. ***p < 0.001; ****p < 0.0001. The significance of the Mukteswar- and JS/MukHN-infected groups was compared to that of the JS/7/05/Ch-infected group.

The velogenic field isolate JS/7/05/Ch originated from the ND mesogenic vaccine strain Mukteswar and the two strains display notable aa differences in their HN proteins. As depicted in Figure 1A, six aa variations were detected in their HN proteins. Utilizing a reverse genetics system, our laboratory previously engineered a chimeric NDV designated JS/MukHN by incorporating genetic material from these two parental viruses, specifically focusing on the replacement of the HN gene. Selecting these three NDVs as models, they were cultured in allantoic cavities and collected 48 h (h) after inoculation for further analysis [21]. Afterwards, the median tissue culture infective dose (TCID_50_) was utilized to assess the viral titres in primary chicken embryo fibroblast (CEF) cells following the standard procedure [22].The DF-1 cell line, an immortalized chicken embryo fibroblast cell line expressing GFP-RFP-LC3, was generously provided by Dr Xiulong Xu (Yangzhou University, China). Additionally, we generated a homology model for the NDV HN protein employing the automated mode of SWISS-MODEL software. High-quality Protein Data Bank (PDB) files were selectively screened, and SWISS-MODEL automatically identified a suitable template (PDB ID: 1e8v.1. A) for homology modelling. The three-dimensional (3D) structure was then computed and visualized using PyMol 2.4.0 software. The 145^th^, 266^th^, 494^th^, and 495^th^ aa were displayed, whereas the 19^th^ and 29^th^ aa were omitted due to the incomplete HN crystal structure. These mutations exhibited distinct distribution patterns at the periphery of the HN dimer (Figure 1B).

### Cytopathic observation and cell viability

Cytopathic effects were detected through a cellular morphology assay using a microscope. Briefly, CEF cells plated in 6-well plates were subjected to mock infection or inoculated with NDVs at a multiplicity of infection (MOI) of 1. The cellular morphology of each experimental group was examined under a microscope (100×) at the indicated times. Furthermore, a Cell Counting Kit-8 (CCK-8, Beyotime Biotech) was used to evaluate cell viability in accordance with the manufacturer's guidelines. CEF cells plated in 96-well plates were infected with NDVs at an MOI of 1 for the specified time intervals. Then, the infected cells were incubated at 37 °C for 1 h following the addition of 10 µL of CCK-8 solution. Finally, an enzyme-linked immunosorbent assay (ELISA) reader was used to assess the absorbance of each well at 450 nm.

### Lactate dehydrogenase (LDH) release measurements

Cytotoxicity was quantified by assessing the release levels of LDH using a cytotoxicity assay kit (Beyotime Biotech). Briefly, CEFs seeded in 96-well plates were exposed to NDV at an MOI of 1 for the designated time. Concurrently, control groups were established, including the blank background group, the control sample group, and the maximum enzyme activity group. The reagent to detect LDH release was added to the wells and allowed to incubate for 1 h prior to the commencement of testing. The medium was then collected and centrifuged to remove any cell debris. Then, we added 60 µL of LDH test solution to the supernatant for 30 min (min) at room temperature in the absence of light. Finally, the optical density was determined at 490 nm. The percentage of cytotoxicity was calculated in accordance with the manufacturer’s guidelines.

### RNA extraction and real-time quantitative PCR (RT-qPCR)

CEFs plated in 6-well plates were subjected to mock infection or inoculated with 1 MOI of NDV for 24 hpi. TransZol Up Reagent (TransGen Biotech) and PrimeScript RT Reagent (Takara) were used for the extraction of cellular RNA and the synthesis of cDNA, respectively. The mRNA expression levels of genes associated with autophagy were assessed by RT‒qPCR. Primer design was guided by the methodology outlined in the referenced study [23] and the primers are shown in Additional file 1. Two microlitres of cDNA from each sample was used to evaluate the amplification of different genes by the SYBR Green dye method (TransGen Biotech) following the manufacturer’s protocol. The 2^−ΔΔCT^ method was used to calculate the relative gene expression. The results are presented as the fold change (2^−ΔΔCT^) in log_10_ compared to the control group.

### Western blot

CEFs plated in 6-well plates were subjected to mock infection or exposed to NDVs at multiple MOIs or time intervals. Following infection, the cells were collected and lysed using RIPA buffer containing the proteinase inhibitor PMSF (Beyotime Biotech). A BCA protein assay kit (Beyotime Biotech) was used to assess the overall protein concentration. Protein samples were mixed with 1 × SDS loading buffer (Beyotime Biotech) and then subjected to denaturation at 100 °C for 10 min. Proteins were fractionated using different concentrations of SDS‒PAGE, with equal amounts loaded onto polyvinylidene difluoride (PVDF) membranes (Bio-Rad). Following a 1-h blocking step at room temperature with 5% skim milk, the primary antibodies, which were appropriately diluted, were incubated overnight at 4 °C followed by incubation with the secondary antibodies for 1 h at room temperature. The following primary and secondary antibodies were used in this study: anti-Caspase-3, -8, and -9 (Proteintech); anti-LC3B, anti-p62/SQSTM1 (Sigma-Aldrich); anti-NF-κB, anti-IκB-α (Proteintech); anti-rabbit or mouse secondary IgG (TransGen Biotech); and antibodies against the indicated proteins (Sigma‒Aldrich). The detection process involved incubating the membrane with a chemiluminescent reagent (Thermo Fisher Scientific). Subsequently, the membrane was imaged in a dark room using chemiluminescence reagents (Tanon). ImageJ v1.48 was used to analyse the images (National Institutes of Health).

### DNA fragmentation assay

Typically, DNA strands break between nucleosomes during the process of cell apoptosis, and cell apoptosis can be confirmed by observing the resulting DNA ladder pattern via gel electrophoresis [24]. A DNA fragmentation assay was conducted following the manufacturer’s instructions with a DNA Ladder Extraction Kit (Beyotime Biotech). Briefly, CEF cells plated in 6-well plates were subjected to mock infection or exposed to NDVs for the indicated times. Then, these cells were harvested and subjected to DNA extraction. After purification, the DNA was subjected to electrophoresis in a 1.0% agarose gel at 80 V. The gel was electrophoresed and subsequently stained with ethidium bromide (Sigma‒Aldrich). Finally, a UV transilluminator (Tanon) was used to analyse the DNA ladders.

### TUNEL assay

A One Step TUNEL Apoptosis Assay Kit (Beyotime Biotech) was utilized to detect apoptotic cells using a terminal deoxynucleotidyl transferase UTP nick-end labelling (TUNEL) assay. Initially, CEFs plated in 6-well plates were subjected to mock infection or exposed to NDVs for the indicated times. Following infection, the cells underwent successive steps of washing, fixation, permeabilization, and blocking. A freshly prepared TUNEL reaction mixture was incubated with the cells for 60 min at 37 ℃ in the dark under humidified conditions. The cell nuclei were stained using DAPI reagent (Beyotime Biotech). The cells positive for TUNEL staining were visualized with a fluorescent microscope (Nikon) and the images were subsequently analysed using ImageJ v1.48 (National Institutes of Health).

### Flow cytometry

Annexin V-FITC/PI double staining is a highly sensitive method for quantitatively detecting apoptosis. Early apoptotic cells (Annexin V+; PI−) and late apoptotic cells (Annexin V+; PI+) were differentiated utilizing an apoptosis detection kit (Beyotime Biotech). First, CEFs were either infected with NDVs at an MOI of 1 or mock-infected in 6-well plates and harvested at the indicated times. Second, the cells were subjected to a series of processes involving rinsing, lysing, centrifugation, and subsequent suspension in PBS. Next, the cells were suspended in Annexin V-FITC binding solution and then incubated with Annexin-V and propidium iodide (PI) at room temperature in the dark for 20 min. Finally, the percentage of apoptotic cells was quantified and analysed using a FACSAria SORP flow cytometer (Becton Dickinson).

### Autophagic flux measurement

Autophagic flux was measured through assays targeting autophagosome and autolysosome formation. First, the cells were transfected with GFP-LC3 to analyse autophagosome formation, followed by treatment with NDV at an MOI of 1 at 24 h post-transfection (hpt). Subsequently, these cells underwent a sequence of procedures, including washing, fixation, and staining with DAPI to visualize their nuclei. The coverslips were placed in mounting solution containing PBS supplemented with 50% glycerol. Next, autophagosomes were visualized using a confocal microscope (Leica). The quantification of green puncta within the cells was performed in ten randomly selected fields at a magnification of 100×. Rapamycin and mock treatment were used as the positive and negative controls, respectively. In addition, green fluorescent protein is susceptible to lysosomal proteolysis and rapidly diminishes in an acidic environment, whereas red fluorescent protein (RFP) maintains its fluorescence under acidic conditions. The distinctive orange puncta may indicate autophagosomes, which result from the fusion of red and green fluorescence [25]. Therefore, the proportion of red to orange puncta reflects the level of autophagy. To monitor the formation of autolysosomes, DF-1 cells stably expressing GFP-RFP-LC3 were infected with NDV at an MOI of 1, while control cells were mock-infected. The cells were subsequently examined using a confocal microscope following the precise procedures detailed earlier. The autolysosomes and autophagosomes are represented by red and orange dots, respectively, and were quantified within the cells across ten randomly selected fields (100× magnification). The percentage of red puncta was calculated as described previously [26].

### Pharmacological inhibition assay

For pharmaceutical intervention, the substances were solubilized in dimethyl sulfoxide (DMSO, Beyotime Biotech) and later diluted in complete medium. Z-VAD-FMK (Beyotime Biotech), a pancaspase inhibitor, was used to inhibit apoptosis, CQ (Sigma-Aldrich) was used to suppress autophagy, and pyrrolidinedithiocarbamic acid (PDTC) (Beyotime Biotech) was utilized to hinder NF-κB activity. After a 1-h preincubation period with Z-VAD-FMK, CQ, or PDTC, the cells were then infected with NDV at an MOI of 1. Cells under control conditions received DMSO treatment. The cellular supernatant and cell lysate were collected at the specified time points for subsequent analysis.

### Dual luciferase assay

CEFs seeded in 24-well plates were co-transfected with 500 ng of pNF-κB-luc plasmid and 50 ng of pRL-TK-RL Renilla luciferase plasmid, both of which were generated by our laboratory. The Renilla luciferase plasmid served as an internal control. The cells were subsequently inoculated with NDVs at an MOI of 1 for 24 h. Next, the cells were treated with detection reagent from a dual-luciferase assay kit (Vazyme). The fluorescence intensity of the cellular samples was quantified by an ELISA reader, and the ratio was calculated.

### Nuclear and cytoplasmic protein extraction

CEF cells plated in 12-well plates were exposed to 1 MOI NDVs for 24 h. Subsequently, the cytoplasmic and nuclear proteins were isolated utilizing a Nuclear and Cytoplasmic Protein Extraction Kit (Beyotime Biotech). Then, the expression levels of NF-κB p65/RelA protein (abbreviated as p65) in the isolated proteins were further evaluated by Western blotting. The proteins GAPDH and histone H3 were used as reference markers for the cytoplasmic and nuclear regions, respectively.

### Statistical analysis

The data are presented as the means ± standard deviations of three independent replicates derived from a representative experiment. Either one-way or two-way analysis of variance (ANOVA) was utilized to evaluate the statistically significant differences, with a significance threshold set at *p* < 0.05. The statistical analyses were conducted with GraphPad Prism 7.00 (San Diego).

## Results

### NDVs harbouring diverse HN proteins differentially induce cytopathic damage

To examine the involvement of the mutant HN protein in vitro, we first assessed cellular cytopathy and cell viability following NDV infection. Microscopy revealed that compared with mock infection, NDV infection caused visible cytopathic effects in a time-dependent manner. The predominant cytopathic alterations observed included changes in cell morphology, characterized by distorted cell shapes, cell rounding, vacuolization, and the accumulation and shedding of a substantial quantity of cell fragments. Among the three NDVs, JS/7/05/Ch induced the most rapid and severe cytopathic effects. Specifically, the JS/7/05/Ch-infected group displayed noticeable cytopathic effects at 12 hpi and exhibited complete disintegration at 36 hpi. In contrast, both Mukteswar and JS/MukHN caused nearly identical cytopathic effects, inducing obvious changes in cell morphology at 24 hpi. The three types of NDV-infected cells disintegrated completely and were not significantly different at 48 hpi (Figure 1C).

The CCK-8 assay revealed that compared with mock infection, NDV infection significantly decreased cell viability in a time-dependent manner. Compared with the other two viruses, JS/7/05/Ch caused a more pronounced decrease in cell viability, notably at 12, 24, and 36 hpi. Mukteswar and JS/MukHN both exhibited comparable cell viability throughout the course of infection (Figure 1D). Additionally, the results of the LDH assay showed that compared with mock infection, NDV infection significantly promoted cellular LDH release. Among the three NDVs, JS/7/05/Ch induced significantly greater LDH release than did the other two viruses at 24, 36, and 48 hpi. As expected, Mukteswar and JS/MukHN had similar effects on LDH release (Figure 1E). Therefore, the velogenic variant NDV strain causes more pronounced cytopathic damage and leads to increased mortality, which is attributed to the mutant HN protein.

### NDVs bearing diverse HN proteins induce differential cell apoptosis

**Figure 2:**
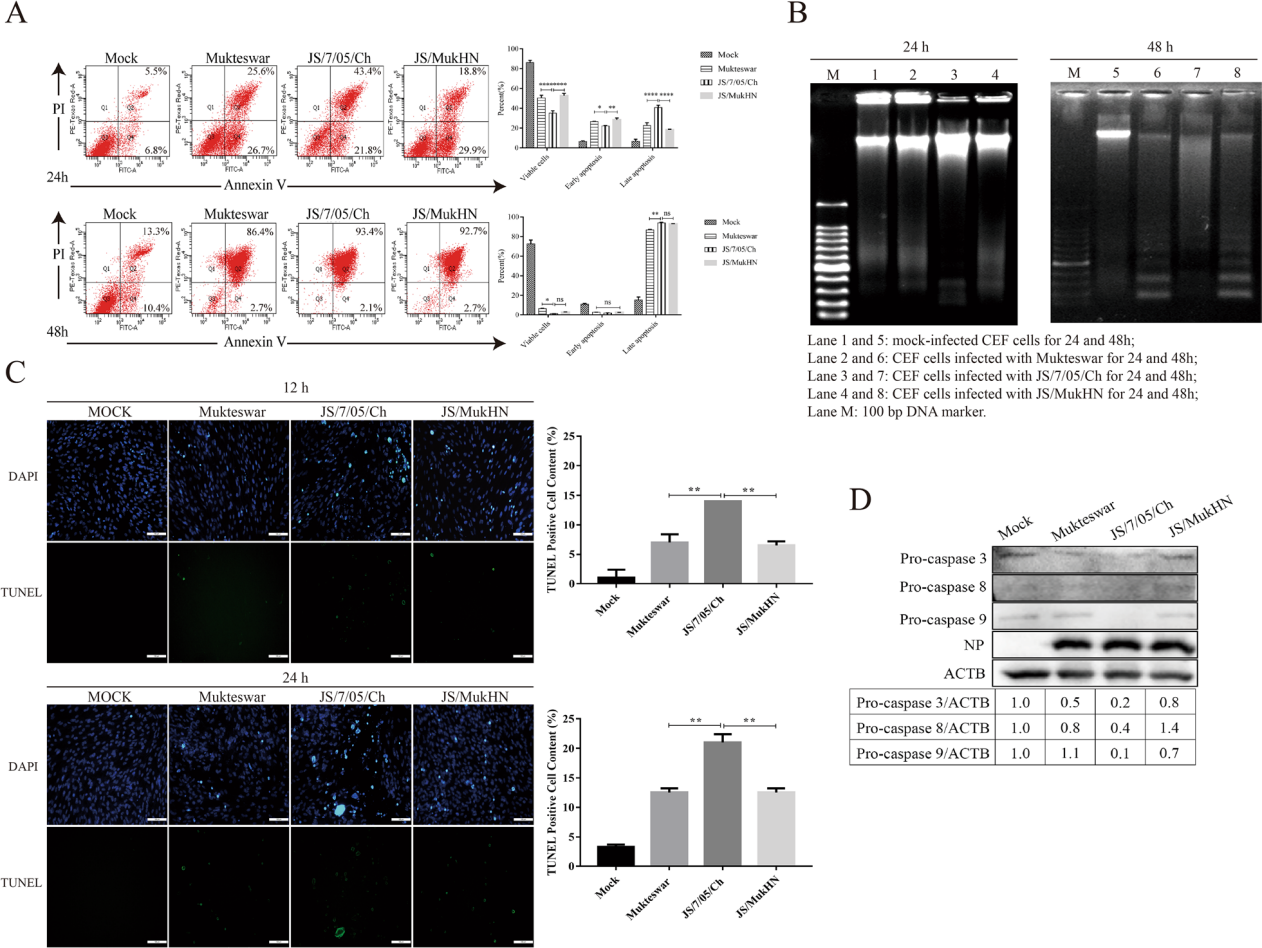
**Assessment of cell apoptosis triggered by NDVs.** **A** The scatter plots depict the flow cytometric assessment of phosphatidylserine (PS) translocation following Annexin V and PI staining in both mock-infected and NDV-infected CEFs at 24 and 48 hpi. The 4 quadrants represent the number of cells in each quadrant. The Q4 quadrant signifies the early stages of apoptosis, while the Q2 quadrant represents late-stage apoptosis and necrosis. The Q3 quadrant is indicative of viable cells. **B** The effects of NDV infection on cell apoptosis were analysed by a DNA ladder assay. Agarose gel electrophoretic patterns showing DNA fragmentation in mock-infected and NDV-infected CEF cells at an MOI of 1. DNA ladders of approximately 180–200 bp of fragmented DNA were visualized by staining with ethidium bromide in a 1.0% agarose gel. **C** The effects of NDV infection on cell apoptosis were analysed by TUNEL assay. After the CEFs were subjected to mock infection or infection with NDV at an MOI of 1 for 12 or 24 h, a quantitative analysis of TUNEL-positive cells was conducted across groups based on a minimum of ten images for each well. The TUNEL-positive cell content was determined by quantifying the number of TUNEL-positive cells relative to the total number of CEFs. Scale bar: 100 μm. **D** The expression levels of apoptotic proteins were detected by Western blotting. Western blotting analysis was performed to determine the protein levels of pro-caspase-3, -8, and -9 in CEFs after mock infection or infection with NDV at an MOI of 1 for 24 h. The data were analysed using ImageJ and are presented as the ratio of pro-caspase protein to the specific ACTB protein. *p < 0.05; **p < 0.01; **** p < 0.0001; ns indicates no significance.

Next, we explored the impact of the mutant HN protein on apoptosis induced by NDV using a variety of assays. The flow cytometric assay revealed a significant increase in the percentage of apoptotic cells among NDV-infected cells compared to mock-infected cells. Among the three NDVs, JS/7/05/Ch induced the highest level of cell apoptosis. At 24 hpi, JS/7/05/Ch induced a percentage of late apoptotic cells of up to 43.4% (Annexin V+/PI+), which was significantly greater than that of Mukteswar (25.6%) and JS/MukHN (18.8%). However, both Mukteswar and JS/MukHN predominantly triggered early apoptosis in cells during this infection period. At 48 hpi, all three NDVs predominantly triggered late-stage apoptosis. JS/7/05/Ch continued to exhibit the most pronounced apoptotic effects at 48 hpi, although the apoptotic disparities among the three infected groups decreased. The difference in late-stage apoptosis levels was notably significant only between JS/7/05/Ch- (93.4%) and Mukteswar- (86.4%) infected cells. As anticipated, Mukteswar and JS/MukHN triggered comparable levels of apoptosis throughout the infection (Figure 2A).

The DNA ladder assay revealed the presence of DNA laddering bands in NDV-infected cells, while such bands were absent in mock-infected cells. Among the three infected groups, evident DNA laddering bands were observed in JS/7/05/Ch-infected cells at 24 hpi, evolving into diffuse bands by 48 hpi. In contrast, clear DNA laddering bands were not induced by Mukteswar or JS/MukHN until 48 hpi (Figure 2B). As revealed by the TUNEL assay, compared with mock infection, NDV infection significantly increased the number of TUNEL-positive cells in a time-dependent manner. Among the three infected groups, the number of TUNEL-positive cells in the JS/7/05/Ch group was greater than that in the Mukteswar and JS/MukHN groups, suggesting a more robust induction of apoptosis during infection. The quantity of TUNEL-positive cells induced by Mukteswar and JS/MukHN was similar (Figure 2C). Thus, these findings illustrate that the velogenic variant NDV strain triggers a heightened level of apoptosis due to the mutant HN protein.

Furthermore, we assessed the expression levels of caspase 3, 8, and 9 to evaluate the extent of apoptosis. Western blot analysis revealed a significant reduction in the expression levels of pro-caspase 3, 8, and 9 following JS/7/05/Ch infection. This suggested that JS/7/05/Ch markedly activated caspase 3, 8, and 9. In contrast, Mukteswar and JS/MukHN exhibited comparatively weaker activation of pro-caspase 3, 8, and 9, indicating a lower level of apoptosis (Figure 2D). Therefore, the velogenic variant NDV strain promotes apoptosis by activating apoptosis-related proteins.

### NDVs harbouring various HN proteins exert distinct modulatory effects on autophagy

**Figure 3:**
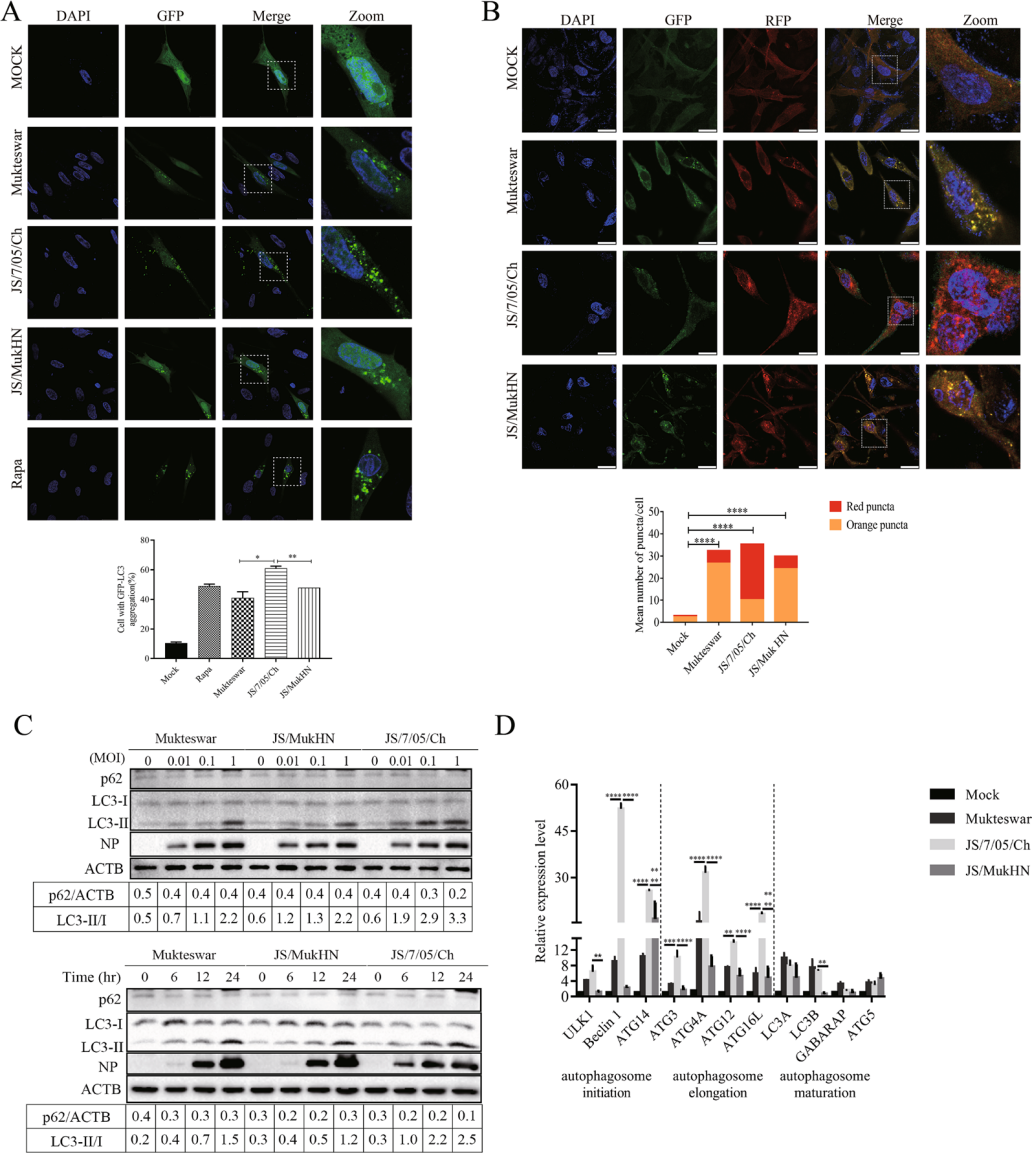
**Evaluation of cell autophagy induced by NDVs.** **A** Determination of autophagosome formation mediated by NDVs. CEF cells transfected with a GFP-LC3 plasmid were exposed to NDVs at an MOI of 1 for 24 h. Subsequently, the cells were examined using fluorescent microscopy to detect the GFP-LC3 puncta. A positive control was established through rapamycin treatment, while a negative control involved DMSO treatment. DAPI (blue) was used for nuclear DNA staining. Scale bar: 20 μm. **B** Determination of autolysosome formation mediated by NDVs. DF-1 cells, genetically modified to express the GFP-RFP-LC3 gene, were subjected to NDV infection at an MOI of 1 for 24 h. Autophagosomes, represented by orange puncta, and autolysosomes, indicated by red puncta, were observed using a confocal microscope. DAPI (blue) was utilized to stain nuclear DNA. Scale bar: 20 μm. The percentage of red puncta was calculated as follows: red puncta (%) = (number of red puncta/(number of red puncta + number of orange puncta)) × 100%. **C** Quantification of autophagy-related protein expression levels after NDV infection at different doses and for different durations. CEF cells were subjected to mock infection or infection with NDVs at multiple MOIs and time intervals. LC3-II lipidation and p62 degradation in cell lysates were evaluated by Western blotting. The data analysed with ImageJ are presented as the ratio of LC3-II to LC3-I and the ratio of p62 to ACTB. **D** The mRNA expression levels of autophagy-related genes in NDV-infected cells. CEF cells were infected with NDV at an MOI of 1 or mock infected for 24 h. Total RNA was extracted from the CEF cells, reverse transcribed, and subjected to qRT‒PCR to measure the relative mRNA expression of autophagy-related genes. *p < 0.05; **p < 0.01; ***p < 0.001; ****p < 0.0001.

Subsequently, we evaluated the impacts of NDV infection on cell autophagy. Transfection of GFP-LC3 protein was used to evaluate the formation of autophagosomes following NDV infection. The intensity and distribution of GFP fluorescent particles can reflect the expression level and intracellular localization of LC3. Confocal fluorescence microscopy showed that in mock-infected cells, GFP-LC3 exhibited a diffuse distribution. In contrast, both the NDV- and rapamycin-treated groups exhibited punctate GFP-LC3 fluorescence, indicating the formation of autophagosomes. Among the three NDV-infected groups, the quantity of GFP-LC3 puncta in JS/7/05/Ch-infected cells was significantly greater (approximately 60%) than that in Mukteswar- and JS/MukHN-infected cells (approximately 40–50%). Furthermore, JS/7/05/Ch-induced GFP-LC3 puncta were perinuclearly distributed, whereas both Mukteswar- and JS/MukHN-induced puncta were mostly scattered on one side near the nucleus. Rapamycin, which was used as a positive control, resulted in an approximately 50% increase in the number of autophagosome-positive cells (Figure 3A). These results indicate that the velogenic variant NDV strain significantly stimulates autophagosome formation, thereby enhancing cell autophagy.

To investigate the impact of viral infection on autophagic flux, we observed autolysosome formation in DF-1 cells expressing GFP-RFP-LC3 upon infection with NDV. Confocal fluorescence microscopy revealed a notable increase in red punctate fluorescence upon NDV infection compared to that in the mock-infected group. Remarkably, JS/7/05/Ch induced a significantly greater percentage of red/orange puncta (exceeding 50%) than did Mukteswar and JS/MukHN (which remained well below 50%) (Figure 3B), indicating that JS/7/05/Ch enhanced autophagic flux. These results suggest that the velogenic variant NDV strain significantly facilitates autophagic flux, thereby boosting the autophagic process.

To comprehensively evaluate the differences in autophagy induced by NDVs, we utilized Western blotting to assess the expression levels of autophagic proteins, specifically LC3 and p62. Compared with mock infection, NDV infection triggered the conversion of LC3-I to LC3-II (also known as LC3-II lipidation). LC3-II lipidation exhibited both dose- and time-dependent effects. Among the three NDV-infected groups, the JS/7/05/Ch-infected group showed greater LC3-II lipidation than the Mukteswar- and JS/MukHN-infected groups, indicating greater autophagy. Furthermore, JS/7/05/Ch efficiently degraded the p62 protein in a dose- and time-dependent manner, whereas neither Mukteswar nor JS/MukHN significantly degraded p62 (Figure 3C). This finding further demonstrated the crucial role of the velogenic variant NDV strain in promoting autophagic flux.

Additionally, the transcriptional levels of autophagic genes, including ULK1, Beclin-1, ATG14 (autophagosome initiation complex), ATG3, ATG4A, ATG12, ATG16L (autophagosome elongation complex), LC3A, LC3B, GABARAP, and ATG5 (autophagosome maturation complex), were assessed by qPCR. The qPCR results revealed that NDV infection can selectively influence the mRNA levels of these autophagy-related genes. Compared with Mukteswar and JS/MukHN, JS/7/05/Ch exhibited significant upregulation of the expression of genes related to the autophagosome initiation complex and elongation complex. Specifically, there were significant differences in the induction of ULK1, Beclin-1, ATG14, ATG3, ATG4A, ATG12, and ATG16L. However, infection with the three NDV strains did not significantly affect the expression of genes related to the autophagosome maturation complex. As expected, the mRNA levels of these genes, which are regulated by Mukteswar and JS/MukHN, were largely consistent (Figure 3D). Taken together, these results suggest that the velogenic variant NDV strain promotes cell autophagy via the mutant HN protein, which is accompanied by enhanced autophagosome formation and increased autophagic flux.

### Apoptosis and autophagy mediate the differential effects of NDV infection on virus replication and cell viability

**Figure 4:**
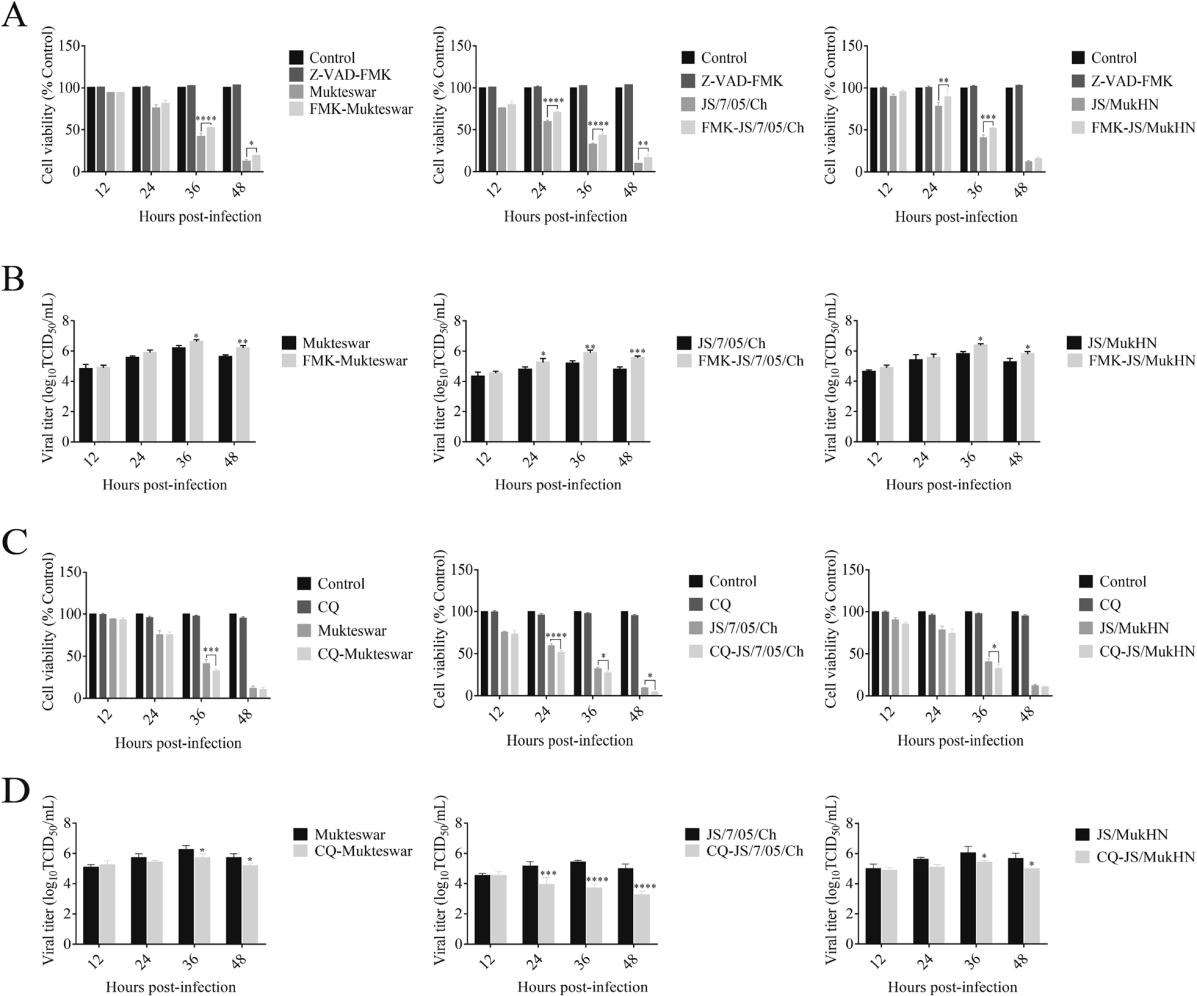
**Impact on cell viability and virus replication after inhibition of apoptosis and autophagy in NDV-infected cells.** CEF cells were incubated with either Z-VAD-FMK (20 µM) for **A** and **B** or CQ (50 µM) for **C** and **D**, while DMSO served as the control. After a 1 h incubation period, the cells were exposed to NDV at an MOI of 1. Subsequently, the cell lysates and supernatants were analysed to assess cell viability (**A** and **C**) and viral titres (**B** and **D**) at 12, 24, 36, and 48 hpi. *p < 0.05; **p < 0.01; ***p < 0.001; ****p < 0.0001.

To explore the function of apoptosis/autophagy in viral infection, we detected cell viability and virus replication after pharmacological inhibition. First, we investigated the impacts of inhibiting apoptosis on cell viability and virus replication in CEF cells pretreated with the apoptosis inhibitor Z-VAD-FMK. Z-VAD-FMK treatment improved cell viability to varying degrees among the three NDV-infected groups. Briefly, the JS/7/05/Ch-infected group exhibited more pronounced changes in cell viability following Z-VAD-FMK treatment. A significant increase in cell viability was observed at 24 (*p* < 0.0001), 36 (*p* < 0.0001), and 48 hpi (*p* < 0.01). In contrast, the impacts of Z-VAD-FMK treatment on cell viability in the Mukteswar- and JS/MukHN-infected groups were consistently less pronounced than those in the JS/7/05/Ch-infected group (Figure 4A). Furthermore, the viral titres of the three NDVs showed varying degrees of upregulation following Z-VAD-FMK treatment. The viral titres of the JS/7/05/Ch-infected group exhibited a significant increase at 24 (*p* < 0.05), 36 (*p* < 0.01), and 48 hpi (*p* < 0.001). In comparison, the Mukteswar- and JS/MukHN-infected groups displayed a less pronounced increase in viral titres than did the JS/7/05/Ch-infected group after Z-VAD-FMK treatment (Figure 4B). Therefore, suppressing apoptosis significantly enhances cell viability and virus replication in NDV-infected cells, particularly when they are exposed to the velogenic variant NDV strain.

Next, we explored the impact of autophagy inhibition on cell viability and virus replication by employing the autophagy inhibitor CQ in CEFs. CQ treatment had varying degrees of impact on cell viability and virus replication among the three infected groups. The CCK-8 results revealed a notable decrease in cell viability within the JS/7/05/Ch-infected group, with significant downregulation observed at 24 (*p* < 0.0001), 36 (*p* < 0.05), and 48 hpi (*p* < 0.05). However, the cell viability of the Mukteswar- and JS/MukHN-infected groups exhibited a notable decrease only at 36 hpi. The magnitude of the reduction in cell viability was less pronounced than that observed in the JS/7/05/Ch-infected group (Figure 4C). Furthermore, the viral titres of JS/7/05/Ch exhibited a significant decrease at 24 (*p* < 0.001), 36 (*p* < 0.0001), and 48 hpi (*p* < 0.0001). In contrast, both Mukteswar and JS/MukHN exhibited similar changes in viral titres after autophagy inhibition, with a significant decrease observed only after 36 hpi. Moreover, the degree of reduction in viral titres was lower in Mukteswar and JS/MukHN than in JS/7/05/Ch (Figure 4D). Therefore, suppressing autophagy markedly diminishes cell viability and hampers virus replication in NDV-infected cells, especially during infection with the velogenic variant NDV strain.

### NDVs bearing diverse HN proteins exhibit distinct modulations of NF-κB activation

**Figure 5:**
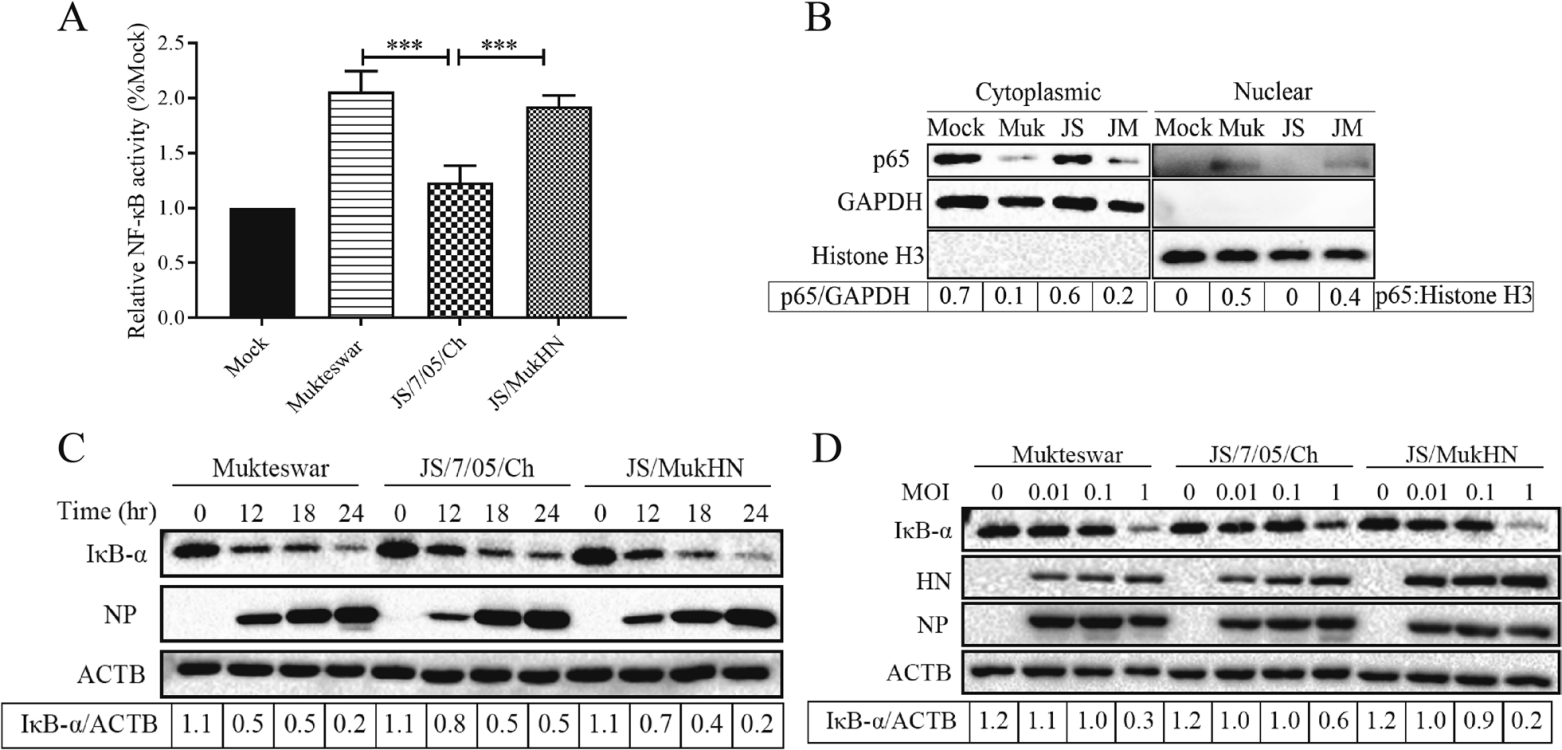
**Assessment of NF-κB activity triggered by NDVs.** **A** NF-κB activity induced by NDVs was assessed using a dual luciferase reporter assay system. CEF cells were co-transfected with luciferase plasmids in 24-well plates, with the Renilla luciferase plasmid serving as a mock control. Subsequently, the transfected cells were exposed to NDVs at an MOI of 1 for 24 h. The relative NF-κB activity in the mock group was considered to be 1. **B** Analysis of the nuclear translocation efficiency of the NF-κB p65 protein. CEF cells were infected with NDVs at an MOI of 1 for 24 h in 12-well plates, and the proteins were then extracted from the cytoplasm and nucleus. The extracted proteins were examined for NF-κB p65 protein levels through Western blot analysis. The cytoplasmic and nuclear proteins were quantified by the GAPDH and histone H3 proteins, respectively. The data analysed with ImageJ are presented as the ratio of p65 to GAPDH in the cytoplasm and the ratio of p65 to histone H3 in the nucleus. Muk indicates Mukteswar, JS indicates JS/7/05/Ch, and JM indicates JS/MukHN. **C** and **D** Assessment of IκB-α protein expression levels after NDV infection in a time- and dose-dependent manner. CEF cells were exposed to NDVs at multiple time intervals (0, 12, 18, and 24 h) and MOIs (0, 0.01, 0.1, and 1 MOI). Then, the protein expression levels of IκB-α were examined by Western blotting. The data analysed with ImageJ are presented as the ratio of IκB-α to ACTB. ***p < 0.001.

To investigate whether NF-κB activation caused differences in apoptosis and autophagy, we assessed the activation of NF-κB following NDV infection. The results of dual luciferase reporter experiments revealed that NF-κB activity was significantly greater in Mukteswar and JS/MukHN than in JS/7/05/Ch (*p* < 0.001). Notably, JS/7/05/Ch did not significantly activate NF-κB (Figure 5A). Under normal circumstances, the NF-κB complex resides in the cell cytoplasm. However, upon external stimulation, NF-κB p65 protein will translocate into the cell nucleus, leading to NF-κB activation. Therefore, we determined the nuclear translocation efficiency of the NF-κB p65 protein. The results of Western blot assays revealed a significant decrease in the levels of NF-κB p65 protein in the cytoplasm of Mukteswar- and JS/MukHN-infected cells. Conversely, the levels of NF-κB p65 protein in the nucleus were elevated, indicating the nuclear translocation of NF-κB. However, JS/7/05/Ch did not elicit notable alterations in NF-κB p65 protein levels within the cytoplasm or nucleus, signifying a low efficiency of nuclear translocation of NF-κB (Figure 5B). These findings suggest that the velogenic variant NDV strain is unable to efficiently induce the nuclear translocation of NF-κB, thereby reducing the activation of NF-κB.

Additionally, the results of Western blot assays showed that NDV infection could induce the degradation of IκB-α, and the extent of degradation was positively correlated with the infection time and dose. All three NDV strains exhibited maximum degradation of IκB-α at 1 MOI and 24 hpi. Importantly, the effect of JS/7/05/Ch on IκB-α degradation was significantly milder than that of Mukteswar and JS/MukHN (Figures 5C and D). Therefore, the velogenic variant NDV strain elicits less severe degradation of IκB-α due to the mutant HN protein, resulting in reduced nuclear translocation and activation of NF-κB.

### NF-κB is essential for NDV-induced apoptosis and autophagy, thereby influencing viral infection

**Figure 6:**
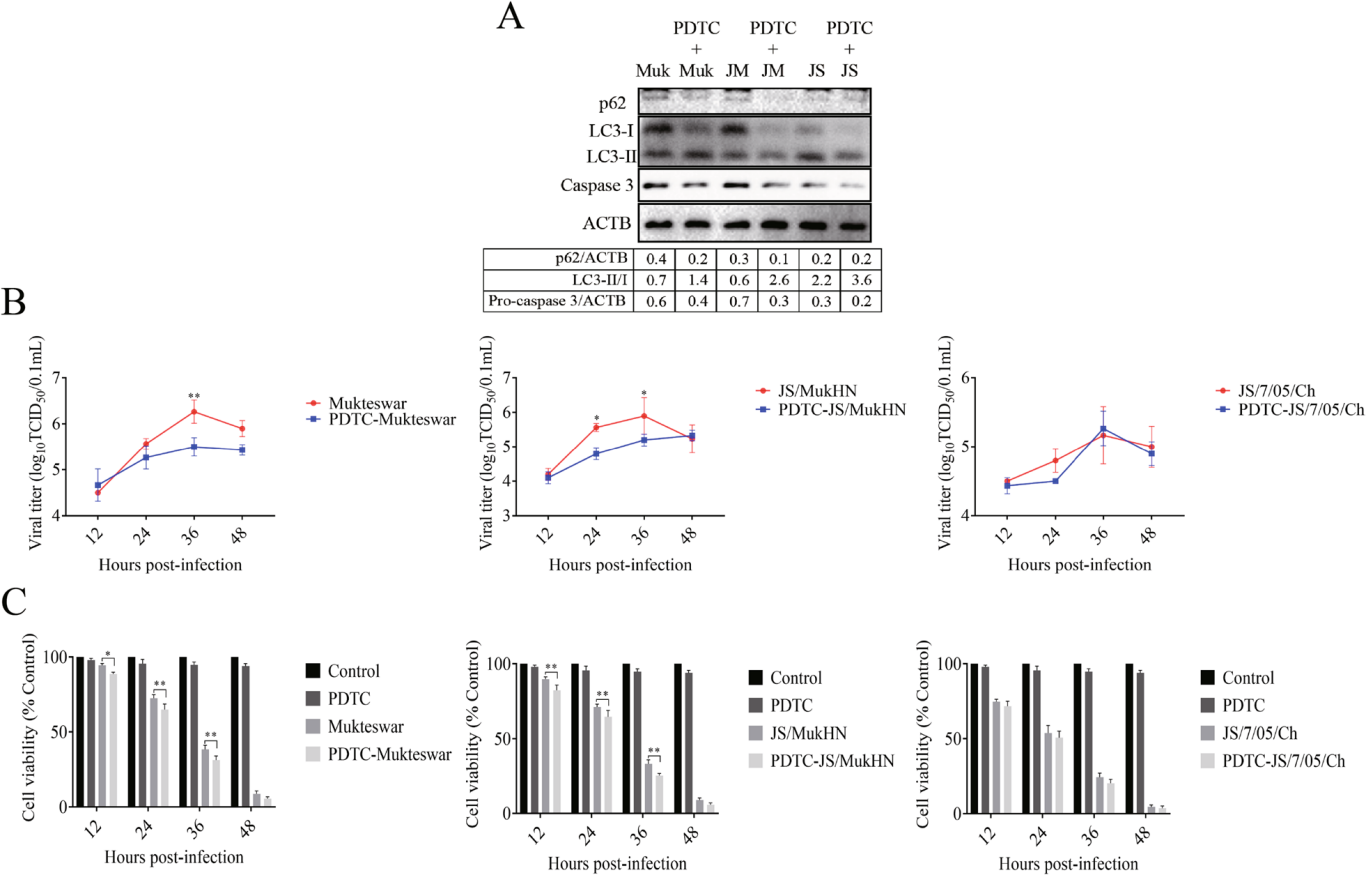
**Effects on apoptosis, autophagy, and viral infection after inhibition of NF-κB in NDV-infected cells.** The NF-kB inhibitor PDTC was used to inhibit NF-kB activity. After preincubation of CEF cells with 60 μM PDTC for 1 h, the cells were infected with NDVs at an MOI of 1 for the indicated time. DMSO treatment served as the control. **A** The cell lysate was collected for the determination of p62 degradation, LC3-II/I conversion, and caspase-3 activation at 24 hpi. The data analysed with ImageJ are presented as the ratios of p62 to ACTB, LC3-II to LC3-I, and pro-caspase-3 to ACTB. Muk indicates Mukteswar, JS indicates JS/7/05/Ch, and JM indicates JS/MukHN. **B** The cell supernatants were analysed to assess viral titres at 12, 24, 36, and 48 hpi through a TCID_50_ assay. **C** The cell lysates were analysed to assess cell viability at 12, 24, 36, and 48 hpi through a CCK-8 assay. *p < 0.05; **p < 0.01.

Since NF-κB is intricately associated with programmed cell death, we explored the impact of NF-κB inhibition on NDV-induced apoptosis and autophagy in CEF cells using the NF-κB inhibitor PDTC. NF-κB inhibition enhanced NDV-induced apoptosis and autophagy, as shown by the activation of caspase 3, degradation of p62, and lipidation of LC3-II. Notably, NF-κB inhibition had a more pronounced effect on the regulation of apoptotic and autophagic proteins in the Muktewar- and JS/MukHN-infected groups. Nevertheless, its influence on JS/7/05/Ch-induced apoptosis and autophagy was relatively limited, especially regarding the activation of caspase 3 and the degradation of p62 (Figure 6A).

To elucidate the impact of NF-κB on viral infection, this study further examined the replication levels of NDV as well as the activity of infected cells following NF-κB inhibition. The results of TCID_50_ assays demonstrated that inhibition of NF-κB significantly reduced the replication levels of Mukteswar and JS/MukHN, whereas its impact on the replication levels of JS/7/05/Ch was not significant (Figure 6B). Similarly, the results of CCK-8 assays revealed a substantial decrease in cell viability in the Mukteswar- and JS/MukHN-infected groups but not in the JS/7/05/Ch-infected group (Figure 6C). Taken together, these findings illustrate that NDVs with various HN proteins can selectively trigger cell apoptosis and autophagy by modulating the activation of NF-κB, consequently impacting the progression of viral infection.

## Discussion

ND presents a significant threat to domestic fowl worldwide, and the primary approach to controlling NDV in poultry is through vaccination [27]. Mukteswar, a mesogenic live vaccine strain for ND, has been extensively utilized in China [28, 29]. Our laboratory previously identified a velogenic variant of the Mukteswar strain, which was specifically designated JS/7/05/Ch. Further research demonstrated that the mutant HN protein is crucial for the enhanced virulence of JS/7/05/Ch [20]. However, its effects on NDV infection in vitro are not clear. Therefore, the objective of this study was to clarify the function of the mutant HN protein in NDV-infected cells.

Generally, virulent NDV can cause cytopathic effects in various cell lines during infection [30, 31]. Here, we established an NDV-infected cell model and found that NDVs harbouring diverse HN proteins induced differential levels of cytopathic damage and cell death. Among the three viruses, the velogenic variant NDV strain JS/7/05/Ch caused the most severe cytopathic effects, which significantly reduced cell viability. Cell damage or death can lead to disruption of the cellular membrane, resulting in the release of enzymes from the cytoplasm. Among these enzymes, LDH exhibits relatively stable enzymatic activity, and the levels of released LDH typically indicate the extent of cell death [32]. Hence, we assessed NDV-induced cell death by quantifying the LDH activity. Consistent with the findings related to cytopathic damage and cell viability, JS/7/05/Ch triggered greater LDH release than the other two viruses. According to these findings, we can tentatively conclude that the mutant HN protein exacerbates NDV-induced cellular damage and death.

Apoptosis, a crucial type of programmed cell death, is a self-regulated cell death process driven by a genetic program [33, 34]. NDV infection can induce cytopathic effects by triggering cell apoptosis. Velogenic NDV strains are particularly advantageous for eliciting early and robust apoptotic responses [35, 36]. Here, JS/7/05/Ch promptly triggered more extensive cell apoptosis by activating caspase proteins in CEFs, which was attributed to the mutant HN protein. These results align with prior research suggesting that the NDV HN protein can trigger cytoplasmic vacuolization, increase caspase levels, disrupt the mitochondrial transmembrane potential, and enhance oxidative stress [37]. Notably, apoptosis appears to directly influence the death of animals infected with highly virulent strains [15]. Consequently, heightened apoptotic levels could contribute to the increased virulence and pathogenicity of JS/7/05/Ch.

Autophagy, a type II programmed cell death pathway, regulates cellular homeostasis by orchestrating the breakdown of impaired cellular components, dispensable proteins, and infiltrating microorganisms [10]. To ascertain whether the mutant HN protein influences NDV-induced autophagy, we systematically assessed autophagic levels following viral infection in CEFs. The experimental results revealed a heightened level of autophagy elicited by JS/7/05/Ch, suggesting that the mutant HN protein amplified NDV-induced autophagy. Autophagy contributes to the regulation of virus replication, creating favourable conditions for successful viral infection [26, 38]. Thus, JS/7/05/Ch increased autophagy, which further facilitated its replication within host cells. Further experiments revealed that apoptosis negatively regulated NDV replication and cell viability, while autophagy positively modulated NDV replication and cell viability. Notably, the mutant HN protein enhanced this effect.

The transcription factor NF-κB is recognized for its pivotal effect in regulating cell survival, apoptosis, and autophagy [39]. Further investigations are needed to elucidate whether the mutant HN protein influences NDV-induced apoptosis and autophagy by modulating NF-κB signalling. Interestingly, the mutant HN protein notably hindered the NDV-induced activation of NF-κB/IκB-α. Moreover, NF-κB had a positive regulatory effect on both NDV replication and cell viability. However, its influence on JS/7/05/Ch was not statistically significant. These results also indicated that the mutant HN protein significantly attenuated the reciprocal interaction between NF-κB activation and NDV infection. The relationship between apoptosis and autophagy is intricate, as autophagy can both facilitate and inhibit apoptosis [40]. Currently, the research indicates that autophagy can promote apoptosis via the NF-κB pathway [41]. Nevertheless, whether this applies to our study remains to be further investigated in subsequent research.

**Figure 7:**
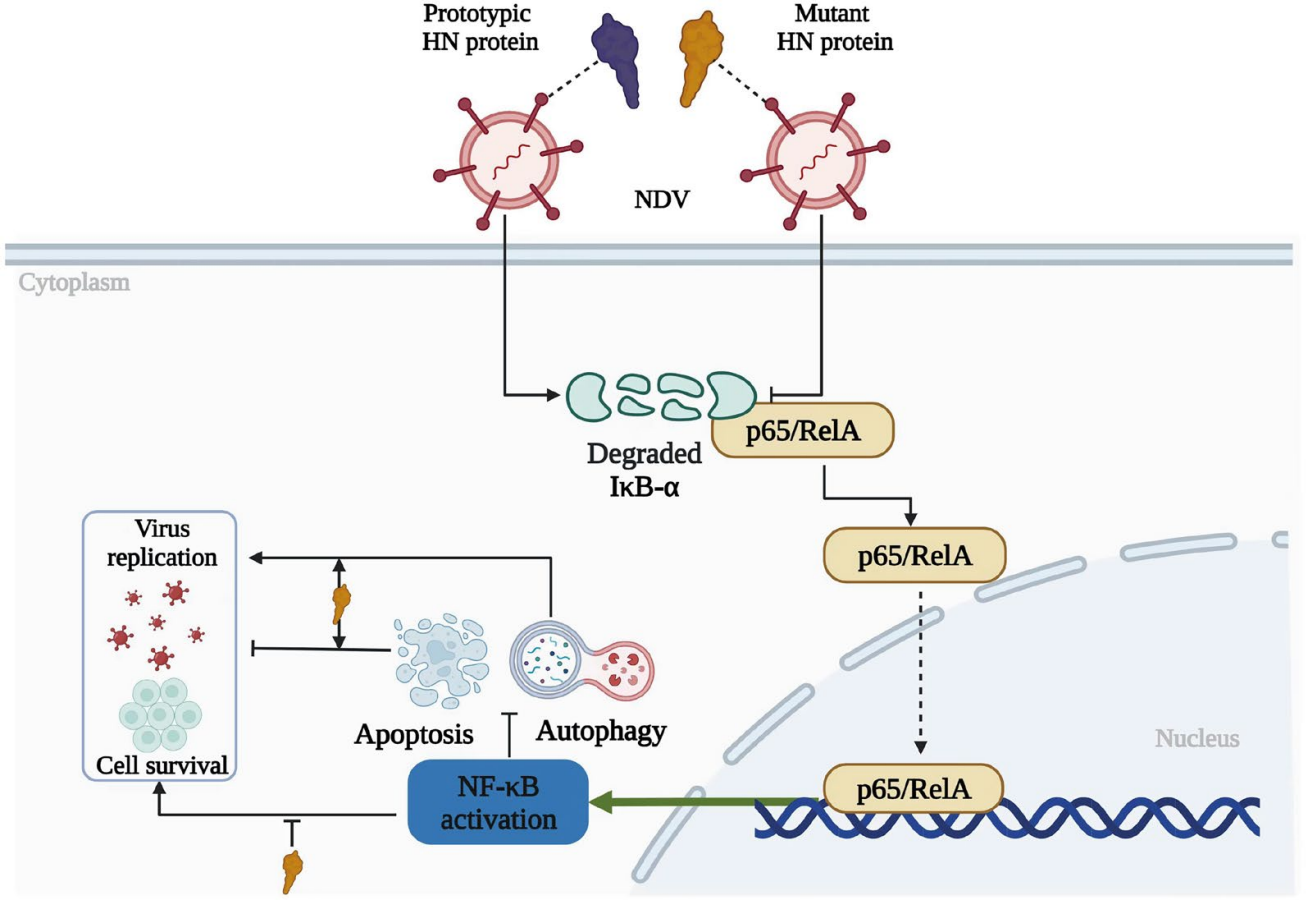
**Schematic diagram illustrating how the mutant HN protein modulates cell apoptosis and autophagy through the NF-κB pathway during NDV infection.** When NDV harbouring the prototypic HN protein infects cells, it can trigger effective degradation of the IκB-α protein, facilitating p65/RelA nuclear translocation and activating NF-κB. This process attenuates cell apoptosis and autophagy but boosts virus replication and cell survival. Conversely, the mutant HN protein weakens the degradation of IκB-α and the nuclear translocation of p65/RelA, thereby inhibiting NF-κB activation during NDV infection. This decrease in NF-κB activation leads to increased apoptosis and autophagy. The mutant HN protein amplifies the effects of apoptosis and autophagy on virus replication and cell survival while impeding the adverse impacts of NF-κB on virus replication and cell survival.

In summary, vaccination is a key approach in poultry management for NDV; however, there is a potential risk of mutation in live vaccine strains. Our laboratory previously isolated a velogenic variant NDV strain derived from the ND vaccine strain that demonstrated heightened virulence due to the mutant HN protein. This study successfully underscores the significance of the mutant HN protein in NDV-infected cells. The mutant HN protein facilitates cytopathic damage and programmed cell death by attenuating the activation of NF-κB in NDV-infected cells (Figure 7). Notably, this study is the first to identify a role of the NDV HN protein in the regulation of apoptosis and autophagy through the targeting of NF-κB. Hence, this investigation delves into the mechanisms behind the heightened virulence of the ND vaccine strain, offering valuable insights for the development of ND vaccines.

### Supplementary Information

**Additional file 1. **qPCR primer sequences for autophagy-related genes.

## Data Availability

All data generated or analyzed during this study are included in this published article.
