# Supplementary material for: The haemagglutinin–neuraminidase protein of velogenic Newcastle disease virus enhances viral infection through NF-κB-mediated programmed cell death
Source: Vet Res. 2024 May 7;55:58. doi: 10.1186/s13567-024-01312-y (PMC11077864; doi:10.1186/s13567-024-01312-y)
Supplement: Supplementary file 1 — Additional file 1. qPCR primer sequences for autophagy-related genes. [file 13567_2024_1312_MOESM1_ESM.docx]

**Additional file 1 The qPCR primer sequences of autophagy related genes**

| Gene name | Sequence | |  |
| --- | --- | --- | --- |
|  | Sense (5'-3') | Antisense (5'-3') | |
| ULK1 | CAGCCCATCCCCAGTGATTT | GCATACCGGAGACTCGAAGG | |
| Beclin1 | TACGCAGGTCAGCTTTGTGT | ACATCATTCTGGCTGGTGGG | |
| ATG14 | GTGCTGACCTGGAAGACTCC | CCAGATCCGTCTCTTCGTCG | |
| ATG3 | GAACGTCATCAACACGGTGAA | TGAGGACGGGAGTGAGGTACTC | |
| ATG4A | CACAGCAGTGCACATTTGCA | CAGAGTCCTGCTGCGTTCCT | |
| ATG12 | GCACCCGCACCATCCA | GAGGCCATCAGCTTCAGGAA | |
| ATG16L | TGCATCCAGCCAAACCTTTC | CGACGCTGGTGGCTTGTC | |
| LC3A | GAATCCCACCCAGGCTTTCT | GTCTCCTGGGAAGCGTAGAC | |
| LC3B | GTACGAGAGCGAGAAGGACG | AGACGGAAGATTGCACTCCG | |
| GABARAP | CTGCGAAGGGGAGAAGATCC | CGCTGTAGGCGATGTAGAGG | |
| ATG5 | ATACAGCCCTTCCTTGGAGC | TAACCCCATCCACAGTTGCT | |
| β-actin | ATTGTCCACCGCAAATGCTTC | AAATAAAGCCATGCCAATCTCGTC | |
